# Inhibition of P-glycoprotein, multidrug resistance-associated protein 2 and cytochrome P450 3A4 improves the oral absorption of octreotide in rats with portal hypertension

**DOI:** 10.3892/etm.2022.11281

**Published:** 2022-03-28

**Authors:** Xiao-Yu Sun, Zhi-Jun Duan, Zhen Liu, Shun-Xiong Tang, Yang Li, Shou-Cheng He, Qiu-Ming Wang, Qing-Yong Chang

Exp Ther Med 12:3716–3722, 2016; DOI: 10.3892/etm.2016.3808

Subsequently to the publication of the above article, an interested reader drew to the authors’ attention that Fig. 2 on p. 3720 contained an apparent error: Namely, the ‘CYP3A4’ and ‘MRP2’ data panels for Group N in Fig. 2C appeared to be overlapping, such that they were derived from the same original source.

The authors have re-examined their original data, and have realized that this figure was inadvertently assembled incorrectly owing to the similarity of the data shown in the two affected panels. The revised version of Fig. 2 is shown on the next page, now including the correct data for the ‘MRP2’ data panel in Fig. 2C. Note that this error did not have a major impact on either the overall results or on the conclusions reported in this study. The authors are grateful to the Editor of *Experimental and Therapeutic Medicine* for allowing them the opportunity to publish this corrigendum. All the authors agree to the publication of this corrigendum, and apologize to the readership for any inconvenience caused.

## Figures and Tables

**Figure 2:**
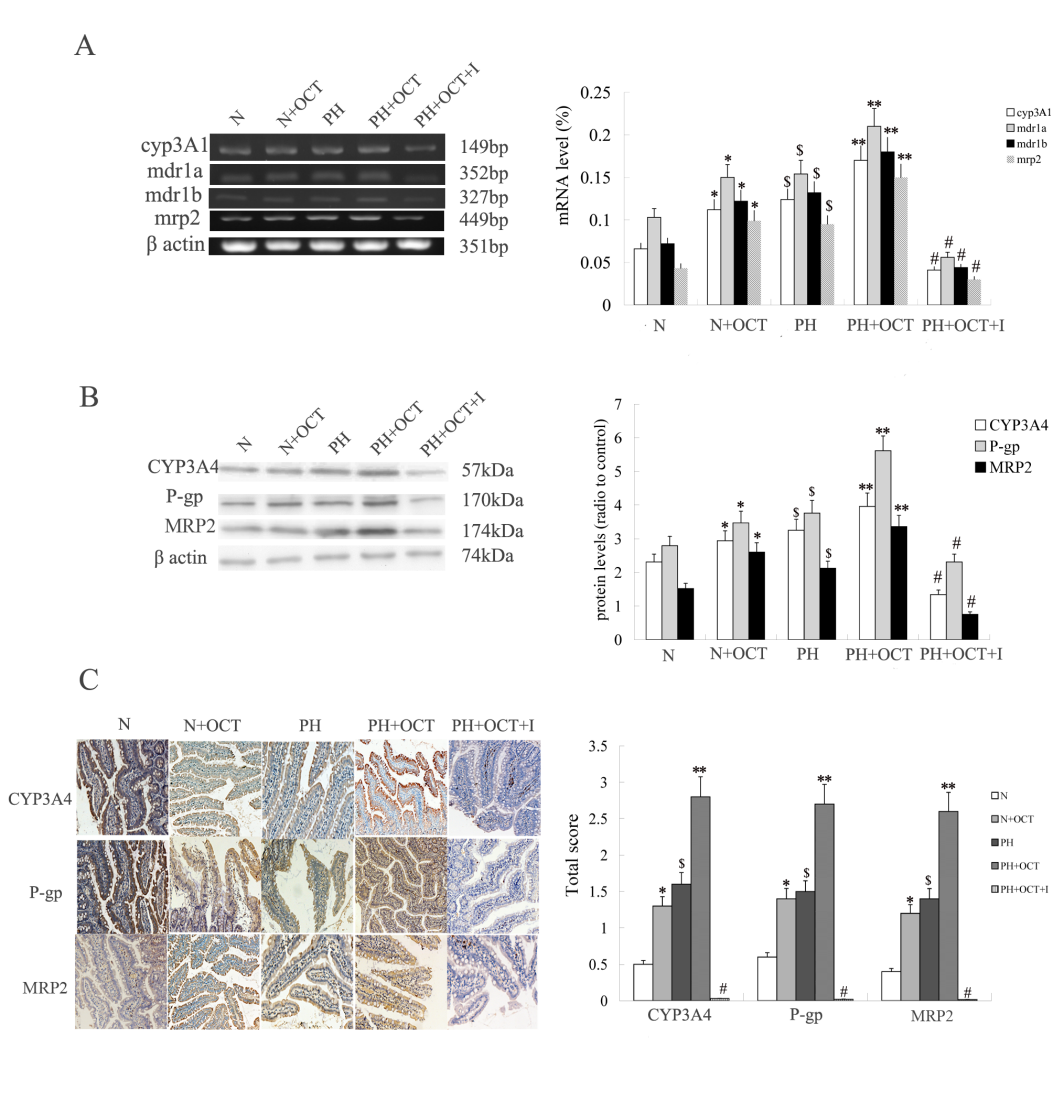
mRNA and protein expression levels of P-gp/MRP2/CYP3A4 in the intestinal mucosa of rats with or without PH. Data are expressed as the means ± standard deviation (n=4). A two-tailed unpaired t-test was used to assess significant differences. (A) Reverse transcription-polymerase chain reaction data showing the CYP3A1, MDR1 (mdr1a, mdr1b) and MRP2 expression levels in the rat intestine in each group. (B) Western blot data showing the CYP3A4, P-gp and MRP2 protein expression levels in the rat intestine in each group. (C) Immunohistochemistry showing the location and extent of CYP3A4, P-gp and MRP2 protein expression in the rat intestine in each group (magnification, x100). ^*^P<0.05, Group N + OCT vs. Group N. ^**^P<0.05, Group PH + OCT vs. Group PH; $P<0.05, Group PH vs. Group N; ^#^P<0.01, Group PH + OCT + I vs. each other group. N, normal control; OCT, octreotide; PH, portal hypertension; I, inhibitors; cyp3a1, cytochrome P450 3A1; MRP2, multidrug-resistence associated protein 2; P-gp, P-glycoprotein.

